# Protective Efficacy of an Inactive Vaccine Based on the LY02 Isolate against Acute *Haemophilus parasuis* Infection in Piglets

**DOI:** 10.1155/2015/649878

**Published:** 2015-11-24

**Authors:** Xiao-Hua Li, Guo-Zhen Zhao, Long-Xin Qiu, Ai-Ling Dai, Wang-Wei Wu, Xiao-Yan Yang

**Affiliations:** ^1^College of Life Science, Longyan University, Fujian Provincial Key Laboratory of Preventive Veterinary Medicine and Veterinary Biotechnology, Fujian Engineering Research Center for Swine Disease Control and Prevention, Longyan, Fujian 364012, China; ^2^College of Animal Science, Fujian Agriculture and Forestry University, Fuzhou, Fujian 350002, China

## Abstract

*Haemophilus parasuis* can cause Glässer's disease characterized by fibrinous polyserositis, polyarthritis, and meningitis. The current prevention of Glässer's disease is mainly based on the inactive vaccines; however, the protective efficacy usually fails in heterogeneous or homologous challenges. Here, the predominant lineage of *H. parasuis* (LY02 strain) in Fujian province, China, characterized as serovar 5, was used to evaluate the protective immunity against acute *H. parasuis* infection in piglets after inactivation. Following challenging with *H. parasuis,* only mild lesions in the pigs immunized with the killed vaccine were observed, whereas the typical symptoms of Glässer's disease presented in the nonimmunized piglets. A strong IgG immune response was induced by the inactive vaccine. CD4^+^ and CD8^+^ T lymphocyte levels were increased, indicating the potent cellular immune responses were elicited. The significantly high levels of IL-2, IL-4, TGF-*β*, and IFN-*γ* in sera from pigs immunized with this killed vaccine suggested that the mixed Th1 and Th2 immune responses were induced, associated with the high protection against *H. parasuis* infection compared to the nonimmunized animals. This study indicated that the inactivated LY02 strain of *H. parasuis* could serve as a potential vaccine candidate to prevent the prevalence of *H. parasuis* in Fujian province, China.

## 1. Introduction

The Gram-negative and NAD-dependent* Haemophilus parasuis* is normally isolated from the upper respiratory tract of healthy swine [1, 2]. The bacteria is also opportunistic pathogen that can lead to severe systemic infection characterized by fibrinous polyserositis, polyarthritis, and meningitis in piglets, known as Glässer's disease [2, 3]. Under the modern intensive production system, this disease, as important emergence, has produced significant mortality and morbidity in pig industry, resulting in severe economic losses worldwide [1, 2, 4].

For controlling Glässer's disease, the primary alternative is considered using vaccination [5]. Although multiple recombinant subunit vaccines have been well evaluated, the currently commercially available vaccines are also mainly based on the inactive component [5, 6]. Several previous studies indicated that the killed vaccines could elicit efficient protective immunity against* H. parasuis* infection compared to any single antigen [5, 7, 8].

So far 15 different serovars of* H. parasuis* have been described. But for epidemiological studies, about 15%–41% of field isolates are nontypeable by serotyping [1, 9]. In China, the prevalence of* H. parasuis* is flourishing, and the most frequent isolates are serotypes 4 and 5 [10]. There are considerable evidences to reveal that the species are very heterogeneous in nature [11, 12], even with the same serotype. The commercial vaccines using* H. parasuis* serotypes 4 and 5 in China thus cannot usually elicit efficient protection against heterogeneous or even homologous challenges, due to limit in cross-protection [13].

In order to identify a novel candidate strain that could elicit efficient immune protection against homologous challenges, various immune responses induced by inactivated* H. parasuis* LY02 strain were examined. Meanwhile, the clinical and pathological lesions of the immunized and nonimmunized piglets after challenge were also evaluated.

## 2. Materials and Methods

### 2.1. Animals

A total of 22 male Landrace × Large White colostrum-deprived (CD) piglets, aged 15 days, were used in the present study and handled in strict accordance with the Good Animal Practice requirements of the Animal Ethics Procedures and Guidelines of China. All the pigs were detected to be negative for* H. parasuis* in both pathogenic and serological tests by the PCR [1] and ELISA [14] methods, respectively.

### 2.2. Bacterial Strains and Growth Conditions

The LY02 strain of* H. parasuis,* isolated from a diseased pig on a farm in Fujian province, was the predominant lineage in this area and was serotyped as serovar 5 using the methods of gel diffusion (GD) and indirect hemagglutination (IHA), following the previous studies [10, 15]. The tryptone soya agar (TSA) and tryptone soya broth (TSB) medium, supplement of a final concentration of 10% horse serum, 5% yeast extract (Becton, USA), and 0.05% NAD (Roche, China), were used to culture the* H. parasuis,* at 37°C in 5% CO_2_.

### 2.3. Preparation of the* H. parasuis* Inactive Vaccine

The* H. parasuis* LY02 strain was serially passaged in the TSB medium to maintain the activity of the bacteria for three times, and the cultured condition was at 37°C, 180 rpm for 18 h. The bacteria were then harvested in PBS to produce a suspension at a concentration of 5 × 10^9^ colony-forming units (CFU) per mL. The suspension was inactivated by treatment with 0.4% formaldehyde for 24 h at 37°C and was then tested by growth on the TSA medium at 37°C for 24 h. The inactivated* H. parasuis* was homogenized with adjuvant in the ratio of 1 : 1.5 (Montanide IMS 2215 [Seppic Inc., Paris, France]) to generate a stable oil-in-water emulsion.

### 2.4. Immunization and Challenge

Piglets were randomly assigned to 4 groups. Group I (G1) and group II (G2) were intramuscularly immunized with 2 mL of the inactivated vaccines, respectively, and given similar booster vaccination 21 days later. The piglets from group III (G3) and group IV (G4) received 2 mL of PBS plus adjuvant. Three weeks after the second inoculation, piglets in group I and III were challenged intraperitoneally with the LY02 strain of* H. parasuis* at the concentration of 7.5 × 10^9^ CFU/mL.

### 2.5. Clinical and Pathological Examination

Rectal temperatures and clinical symptoms of piglets after immunization were assessed daily until the end of the study. All the animals were subjected to necropsy, and the gross lesions especially in the organs of pleural, pericardial, and peritoneal cavities, the hock, carpal, and stifle joints, and lungs were recorded. The tissue samples were obtained for bacterial isolation and histopathological examination.

### 2.6. Bacterial Isolation

Specimens from lymph, heart, lungs, liver, spleen, brain, and kidneys and swabs from the pleural, pericardial, and peritoneal cavities and from stifle joints were used for bacterial isolation when animals died after the challenge. The tissues from survival pigs were collected at twenty days after challenge. All the samples were collected aseptically and were incubated using TSA medium as conditions described above. The colonies were then identified by Gram stain and PCR [16].

### 2.7. Histopathological Examination

Specimens of lymph nodes, lungs, heart, spleen, liver, and kidneys were collected in 10% neutral-buffered formalin, embedded in paraffin wax, sectioned at 3 mm, and stained with haematoxylin and eosin (HE).

### 2.8. Antibody Assays

Blood samples of pigs from each group were collected from the precaval vein at days 0, 21, 35, 42, 49, 56, and 70. Then the sera were obtained by centrifugation of blood samples at 2500 rpm for 25 min. The specific antibodies were detected by indirect ELISA, following the instructions of the manufacturer (FEIKAI, Biotech Co., Ltd., Beijing, China). Briefly, each well of 96-well microtiter plates was coated with soluble antigens of* H. parasuis*. The nonspecific binding sites were blocked with TBS (150 mmol/L NaCl, 10 mmol/L Tris-HCl) containing 5% bovine serum albumin. The tested serum samples were added to the wells and incubated at 37°C for 1 h. Each well was then incubated with HRP-conjugated anti-swine IgG (diluted 1 : 250). After adding 200 *μ*L substrate solution (80 *μ*g of 3,3′,5,5′-tetramethylbenzidine, 30% H_2_O_2_), the reaction was stopped with 2 M H_2_SO_4_. All measurements were made in triplicate at an absorbance of 450 nm.

### 2.9. Cytokine Assays

The sera from piglets in each group were also used for assay of titers of IL-2, IL-4, IL-10, TGF-*β*, and IFN-*γ* using commercial ELISA kits according to the manufacturer's instructions (Bogoo, Biotech Co., Ltd., Shanghai, China). The analysis was performed on the data from three independent experiments.

### 2.10. Flow Cytometry

The collected blood samples were also used to determine the percentage of CD4^+^ and CD8^+^ T cells after purification by removing the red blood cells using RBC lysis solution (BD Biosciences, USA). Then the lymphocytes were stained with Alexa Fluor- (AF-) labeled CD3 MAb (BD Biosciences), phycoerythrin- (PE-) labeled CD4 MAb (BD Biosciences), and fluorescein isothiocyanate- (FITC-) labeled CD8 MAb (BD Biosciences) antibodies. After washing with PBS, the cells were fixed with 5% paraformaldehyde solution in PBS containing 1% BSA plus 0.1% sodium azide. All the samples were analyzed by fluorescence profiles on an FACScan flow cytometer (BD Biosciences) using SYSTEM II software (Coulter).

### 2.11. Statistical Analysis

Data regarding antibody responses, lymphoproliferation assays, cytokine production, and percentages of CD4^+^ and CD8^+^ T cells were statistically analyzed by the procedure of SAS (Statistical Analysis System, version 8.0) using the method of one-way ANOVA. The level of significant difference in comparisons between groups was defined as *P* < 0.05.

## 3. Results

### 3.1. Clinical Evaluation

Clinical symptoms in piglets from G3 were observed at 7 h postinfection (PI) that included prostration and lassitude. The temperatures of all challenged pigs were shown between 39.4 and 41.0°C and reached the highest level at 48 h PI (*P* < 0.01). The temperatures in the pigs immunized with the inactive vaccine were returned to normothermia at 4 days PI (Table 1). The clinical symptoms in pigs from group I were observed in several members at 30 h PI and gradually disappeared at 72 h PI. All the pigs in group III were dead at 50 h PI, with additional clinical findings of incoordination, ataxia, anorexia, severe dyspnoea, and coughing. No clinical signs were recorded in these piglets from groups II and IV during the observation until the end of the experiment at 10 days PI.

### 3.2. Protection of Vaccinated Pigs

To evaluate the protective immunity of this inactive vaccine, 21 days after the last immunization, pigs from each group were average-challenged with a lethal dose of LY02 strain. All the pigs in the negative control groups were dead at about 50 h PI (Table 2). Meanwhile, the pigs immunized with inactive vaccine survived until the end of the observation. Immunization of the vaccines including LY02 strain antigen significantly increased the survival rate statistically (*P* < 0.05). Twenty days after challenge, several tissues from pigs in each group were collected to isolate* H. parasuis*. The bacteria were identified in all samples taken from G3 animals but were not detected in pigs from G1, G2, and G4.

### 3.3. Gross Pathological Changes

**Figure 1:**
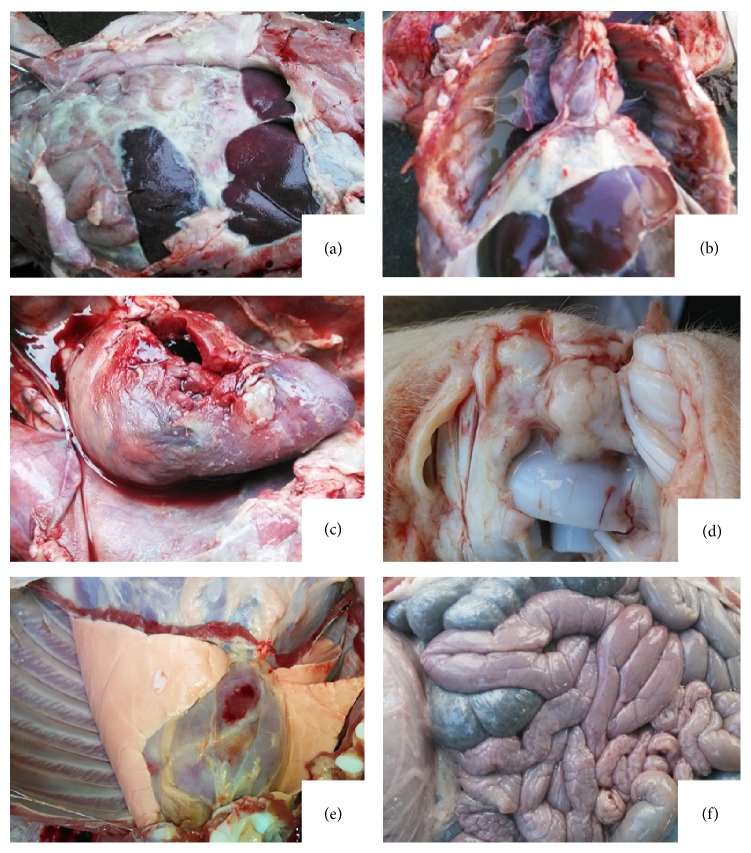
Gross necropsy findings of challenged piglets. (a)–(d) Fibrinous layers on serosal surfaces in enterocoelia, thorax, pericardium, and articular cavity from nonvaccinated pigs. (e) and (f) Lesions in thorax and enterocoelia from immunized pigs.

The only lesions in the vaccinated pigs after being challenged with* H. parasuis* were observed occasional mild pneumonia. However, the gross lesions were severe in the dead animals from G3. The characteristic polyserositis mixed inflammatory exudate was observed in the articular cavity and pericardial, pleural, and peritoneal cavities with the changes of fibrin strands or layers on serosal surfaces (Figure 1). Spleen and inguinal lymph nodes were enlarged. In the lungs, lesions of exudative pneumonia characterized by the appearance of serofibrinous transudation, alveolar oedema, and hyperemia were observed (Figure 1).

### 3.4. Histopathological Examinations and Bacteriologic Findings

**Figure 2:**
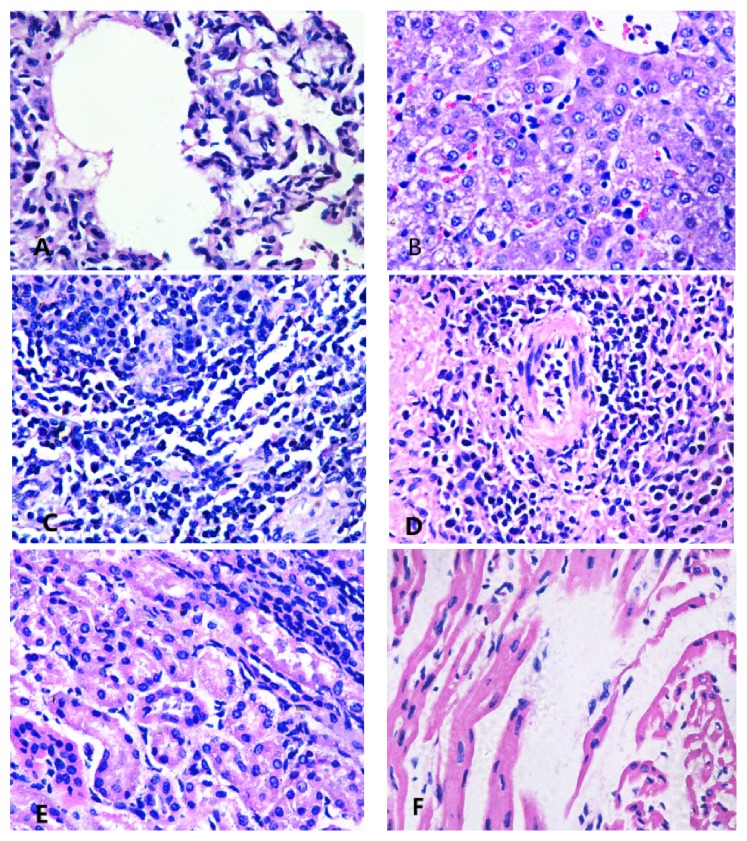
Histopathological examinations in various organs from nonvaccinated pigs. (A) Lung, interstitial pneumonia, inflammatory cells infiltration, alveolar sacs expansion, and alveolar exudate. (B) Liver, cells degeneration, sinus gap expansion, a fibrinous exudate, and focal necrosis foci. (C) Lymph, necrocytosis. (D) Spleen, fibrinous exudate in medullary sinuses, inflammatory cells infiltration, red pulp expansion, and white pulp atrophy. (E) Kidney, epithelial cells necrosis, and lumen with fibrinous exudate. (F) Heart, cardiac muscle fiber atrophy, and flat, myocardial fibers cytoplasm dissolution and fracture.

The histopathological findings appeared hyperaemic, with petechiae or ecchymoses in the cardiac, coronary sulcus, liver, lungs, and brain. The mediastinal and mesenteric lymph nodes appeared hyperaemic and enlarged. Necrocytosis and cytolysis were detected in spleen, heart, kidney, and lymphonodus (Figure 2).

### 3.5. Evaluation of the Humoral Immune Responses

The specific antibodies against* H. parasuis* were evaluated by ELISA. The level of IgG in pigs from G1 was increased with the immunization and reached the significant highest level at the 2 weeks PI compared to the controls (*P* < 0.05). The antibody level was not statistically decreased until 28 days, shown in pigs from G2 (*P* > 0.05) (Table 3). However, the antibody titers were not increased following being challenged with* H. parasuis* LY02 strain in nonvaccinated pigs until all were dead.

### 3.6. Cytokine Production

Two weeks after the final immunization, cytokines of IL-2, IL-4, IL-10, TGF-*β*, and IFN-*γ* in sera were also detected. The highest level of IL-2 was examined in sera of pigs from G2 after the second immunization compared to that in controls (*P* < 0.001, Table 4). The IL-2 level in vaccinated pigs was significantly higher than that in pigs from G3 before acute challenge (*P* < 0.001). The IL-4 titers in pigs from G1 and G2 were maintained at a higher level than that from G3 since the first immunization (*P* < 0.001, Table 4), lasting to all controls which are dead. A significant high level of TGF-*β* and IFN-*γ* was observed in serum samples from pigs in immunized group after the second vaccination compared with that in the controls (*P* < 0.001). However, the level of IL-10 was not significantly increased with successive immunizations compared to that in the controls (*P* > 0.05) (Table 4).

### 3.7. Percentages of CD4^+^ and CD8^+^ T Lymphocyte

As shown in Table 5, the percentages of CD3^+^ CD4^+^ CD8^−^ (*P* < 0.01) and CD3^+^ CD4^−^ CD8^+^ (*P* < 0.001) T lymphocytes in pigs from G2 were significantly lower than those from G1, G3, and G4, and the highest was found in pigs from G1. The ratio of CD3^+^ CD8^+^/CD3^+^ CD4^+^ T cells in the blood samples of pigs from G3 was significantly lower than those from G1, G3, and G4 (*P* < 0.01).

## 4. Discussion

In recent years, the emergence of* H. parasuis* in China has been associated with a wide range of diseases in pigs, usually with high morbidity and mortality [2, 17, 18]. However, no effective vaccines and sensitive diagnostic methods could be available to control the prevalence of the bacteria because more than 15 serovars have been identified [11]. As we know, there are no commercial vaccines with a significant effect against all serovar strains. Screening of novel strains as vaccine candidates is thus of rather a desire.

Several previous studies have shown that the inactive vaccines against* H. parasuis* could elicit better immune protection compared to subunit vaccines [5, 7]. Here, immunization of piglets with the inactivated* H. parasuis* LY02 strain could induce strong humoral and cellular immune responses associated with the increasing survival rate (100%), which would be used for development of new inactive vaccines.

After being challenged with* H. parasuis* LY02 strain, most of the clinical signs and lesions in the nonimmunized piglets presented as typical symptoms of Glässer's disease and were similar to the descriptions in previous studies [5–7]. However, only the appearance of exudates of plasma proteins (fibrin deposits) in the marginal zone of white pulp and in red pulp in spleens in piglets challenged with* H. parasuis* Nagasaki strain [19] was inconsistent with the present study.

Previous study indicated that the specific antibody against* H. parasuis* has no effects on killing the bacteria due to the capacity of bacteria to resist the bactericidal activity of the host complement [20, 21]. However, in this study, the pigs immunized with inactive vaccines did increase the survival rate and decrease* H. parasuis* loading. We speculated that, in the total IgG antibodies, IgG2a was the predominant subclass that has been shown to be the most effective at binding to FcgammaRI on phagocytic cells [22]. As we know, a higher level of serum IgG2a, associated with a Th1-type response, is effective at mediating bacterial opsonophagocytosis [23]. The subclasses of IgG should be further detected.

The cell-mediated immunity plays an important role in mediating resistance to intracellular organisms infection where both CD4^+^ and CD8^+^ T cells are responsible for the control of development and spread of pathogenic bacteria infections [24, 25]. In the present study, the increased levels of CD4^+^ and CD8^+^ T cells in the pigs immunized with the inactive vaccine contributed to the strong protective immunity, consistent with previous studies [5, 6, 26].

Cytokine expression is usually associated with systemic responses, which could also reflect the phenotype of immune response (Th1 or Th2) [27]. IL-2, IFN-*γ*, and TGF-*β* are the Th1-biased cytokines, which can activate the phagocytosis of macrophage, crucial for the resistance to* H. parasuis* infection. The cell-mediated immunity, especially the Th1 type cells, plays the important role in protection against the* H. parasuis* infection. Another crucial role of IFN-*γ* in the immune response is that it regulates differentiation of naïve CD4^+^ T cells to Th1 cells. The IL-4 and IL-10 are the predominant Th2-type cytokines, contributing to the activation of B lymphocytes [22, 26, 28]. Here, the increased IL-2, IL-4, IFN-*γ*, and TGF-*β* cytokines in the vaccinated pigs compared to those in controls indicated that the mixed Th1 and Th2 immune responses were induced, which would be associated with the high protection against* H. parasuis* infection.

## 5. Conclusion

In conclusion, pigs immunized with the inactivated LY02 strain of* H. parasuis* could induce potent humoral and cellular immune responses and develop true protective immunity against the bacteria infection. This study suggests that the inactivated LY02 strain of* H. parasuis* could serve as potential vaccine candidates to prevent the prevalence of* H. parasuis*, at least in Fujian province, China, and also allows us to further analyze the protective efficacy against heterologous challenge.

## Figures and Tables

**Table 1:** Temperatures (°C) in survival piglets at intervals postinfection.

Group	Temperatures (mean ± SD) at intervals after challenge				
	0 h	24 h	48 h	72 h	96 h
G1 (*n* = 7)	39.4 ± 0.3 (7)	40.4 ± 0.6 (7)	40.8 ± 0.2^*∗∗*^ (7)	39.9 ± 0.3 (7)	39.6 ± 0.2 (7)
G2 (*n* = 4)	39.5 ± 0.2 (4)	39.4 ± 0.4 (4)	39.5 ± 0.3 (4)	39.7 ± 0.3 (4)	39.6 ± 0.2 (4)
G3 (*n* = 5)	39.6 ± 0.1 (5)	40.7 ± 0.3 (2)	41.5^*∗∗*^ (1)	—	—

The pig groups are defined in Section 2.

The number of survival pigs is shown in parenthesis.

^*∗∗*^ *P* < 0.01 compared to the controls.

**Table 2:** The protective effect of piglets in each group at 21 days after the second immunization.

Group	Number of animals	Challenged dose (CFU)	Number of animals with clinical signs^a^	Morbidity (%)	Survival (%)	Survival time^b^ (d)
G1	7	7.5 × 10^9^	4	1.43	100	10 ± 0.0
G3	5	7.5 × 10^9^	5	100	0	1.9 ± 0.8
G4	4	0	0	0	100	10 ± 0.0

^a^The assessed clinical signs included incoordination, ataxia, anorexia, severe dyspnoea, and coughing.

^b^The days of survival after challenge for each piglet were recorded until 10 days.

**Table 3:** Levels of IgG antibody in the sera of piglets from each group.

Group	Number of animals	Levels of IgG antibody (means ± SD) at intervals (days)						
		0	21	35	42	49	56	70
G1	7	1.6 ± 1.2	1.4 ± 0.7	1.3 ± 0.2^*∗*^	1.1 ± 0.2	3.9 ± 1.8^#*∗∗*^	5.1 ± 0.8^#^	3.9 ± 0.6^#^
G2	6	1.9 ± 1.4	1.5 ± 0.4	1.3 ± 0.2^*∗*^	1.4 ± 0.1^*∗*^	1.5 ± 0.5	1.3 ± 0.2	1.4 ± 0.3
G3	5	1.2 ± 0.9	0.9 ± 0.5	0.8 ± 0.1	0.9 ± 0.2	0.9 ± 0.3	—	—

^*∗*^ *P* < 0.05, ^*∗∗*^ *P* < 0.01 compared to G3.

^#^ *P* < 0.001 compared to G2.

**Table 4:** Levels of various cytokines in the sera of piglets from each group.

Cytokine type	Group	Levels of each cytokine (means ± SD) at intervals (days) after the immunization				
		21	35	42	49	56
IL-2	G1	104.5 ± 7.4	124.1 ± 19.5	116.1 ± 23.6	125.0 ± 15.5^*∗∗∗*^	113.5 ± 28.3
	G2	122.2 ± 16.5	144.5 ± 22.4^*∗∗*^	111.3 ± 7.6	102.0 ± 19.6^*∗∗∗*^	101.2 ± 23.3
	G3	105.7 ± 22.5	97.6 ± 23.5	90.3 ± 16.5	46.4 ± 19.5	—

IL-4	G1	508.8 ± 52.0^*∗∗*^	540.1 ± 26.5^*∗*^	512.3 ± 66.0^*∗*^	661.5 ± 81.0^*∗∗∗*#^	453.4 ± 68.4
	G2	465.5 ± 40.3	508.0 ± 64.5	500.0 ± 22.3^*∗*^	445.5 ± 36.3^*∗∗*^	456.2 ± 114.9
	G3	378.5 ± 10.4	428.0 ± 23.6	345.0 ± 52.1	191.8 ± 94.9	—

IL-10	G1	408.3 ± 113.5	397.7 ± 106.7	413.8 ± 154.2	462.1 ± 123.5	423.3 ± 134.1
	G2	376.5 ± 143.2	413.8 ± 118.8	409.1 ± 114.5	496.0 ± 103.5	483.5 ± 164.3
	G3	352.6 ± 122.3	326.9 ± 159.6	299.7 ± 163.8	342.9 ± 145.2	—

TGF-*β*	G1	153.4 ± 50.2	173.5 ± 42.6	216.4 ± 30.8	197.5 ± 27.7^*∗∗∗*^	203.3 ± 30.4
	G2	126.8 ± 34.6	168.7 ± 23.4	176.5 ± 32.4	183.6 ± 19.2^*∗∗∗*^	195.5 ± 27.5
	G3	105.3 ± 53.4	96.2 ± 37.9	124.6 ± 34.8	116.8 ± 14.6	—

IFN-*γ*	G1	82.4 ± 24.3	123.5 ± 54.6	155.9 ± 12.9^*∗∗∗*^	187.6 ± 23.6^*∗∗∗*^	178.1 ± 20.5
	G2	79.7 ± 13.8	103.2 ± 31.5^*∗*^	175.6 ± 31.9^*∗∗∗*^	189.8 ± 14.1^*∗∗∗*^	218.3 ± 27.1
	G3	62.3 ± 15.6	58.7 ± 11.1	73.5 ± 20.9	65.1 ± 21.3	—

^*∗∗*^ *P* < 0.01, ^*∗∗∗*^ *P* < 0.001 compared to G3.

^#^ *P* < 0.001 compared to G2.

^*∗*^ *P* < 0.001.

**Table 5:** The percentages of T cell subclasses in immunized piglets 1 week after the last immunization.

Group	T cell subclasses		
	CD4^+^ CD8^−^ (%)	CD4^−^ CD8^+^ (%)	CD3^+^ CD4^+^/CD3^+^ CD8^+^
G1	14.7 ± 1.50^*∗∗*^	30.0 ± 4.70^*∗∗∗*^	0.72 ± 0.07^*∗∗∗*^
G2	12.9 ± 1.70^*∗*^	29.5 ± 4.60^*∗∗∗*^	0.64 ± 0.10^*∗∗*^
G3	6.6 ± 4.10	62.4 ± 13.40	0.29 ± 0.06
G4	13.7 ± 1.30^*∗∗*^	29.2 ± 5.00^*∗∗∗*^	0.73 ± 0.05^*∗∗∗*^

^*∗*^ *P* < 0.05, ^*∗∗*^ *P* < 0.01, and ^*∗∗∗*^ *P* < 0.001 compared to G3.
